# Evaluation and Validation of the Detection of soluble Triggering Receptor Expressed on Myeloid Cells 1 by Enzyme-linked immunosorbent Assay

**DOI:** 10.1038/srep15381

**Published:** 2015-10-20

**Authors:** Astrid Hasibeder, Pamela Stein, Ricardo Brandwijk, Hansjörg Schild, Markus P. Radsak

**Affiliations:** 1 IIIrd Department of Medicine, Johannes-Gutenberg University Medical Center, Mainz, Germany; 2 Center for Thrombosis and Haemostasis, Johannes-Gutenberg University Medical Center, Mainz, Germany; 3 R&D department, Hycult Biotech, Uden, The Netherlands; 4 Institute of Immunology, Johannes-Gutenberg University Medical Center, Mainz, Germany

## Abstract

Triggering receptor expressed on myeloid cells (TREM)-1 plays an important role in innate immune responses and is upregulated under infectious as well as non-infectious conditions. In addition, a soluble TREM-1 variant (sTREM-1) is detectable in sera or bronchoalveolar-lavage fluids from patients. Currently, various studies are difficult to compare, since the methods of detection by enzyme-linked immunosorbent assays (ELISA) vary among different research groups. In this study, we compared three different s-TREM-1 specific ELISAs and identified individual assay characteristics finding notable differences in sTREM-1 concentrations in part depending on the employed buffers. Investigating potential confounding factors for sTREM-1 detection, serum heat-inactivation (HI) showed improved recovery compared to non-HI (NHI) serum, reproducible by addition of complement and re-heat-inactivation. Hence we identified complement as a heat-sensitive confounder in some sTREM-1 ELISAs. We conclude that it is difficult to directly compare data of several studies, in particular if different ELISAs are engaged. Immunoassays for research use only are in general hampered by lack of standardization. Further standardization is needed until sTREM-1 ELISA is capable for better reproducibility of studies and clinical application.

Triggering receptor expressed on myeloid cells (TREM)-1 is expressed on monocytes/macrophages and neutrophils. As an Ig superfamily cell surface molecule activation is transmitted through the transmembrane adapter protein DNA activating protein 12 (DAP12). Activation results in release of pro-inflammatory chemokines and cytokines, increased surface expression of cell activation markers and degranulation. TREM-1 up-regulation has been initially detected *in vitro* after activation with bacterial or fungal stimuli^1^. Immunhistochemistry confirmed *in vivo* high expression levels of TREM-1 in inflammatory lesions caused by bacteria and fungi, e.g. in folliculitis and impetigo, but not in non-infectious inflammatory processes, such as vasculitis and psoriasis^2^. Beyond this also Marburg and Ebola virus activate TREM-1 on human neutrophils^3^. Later up-regulation of TREM-1 on neutrophils has also been detected in non-infectious conditions like critical limb ischaemia (CLI)^4^, rheumatoid arthritis^5^ and inflammatory bowel disease^6^,^7^ indicating a role for TREM-1 also in non-infectious inflammatory responses. As natural TREM-1 ligands Haselmayer *et al.* describe a ligand for TREM-1 on human platelets demonstrated by specific binding of recombinant soluble TREM-1 on human platelets^8^. Additionally, neutrophil peptidoglycan (PGN) recognition protein 1 (PGLYRP1) has recently been identified as another ligand for TREM-1. Complexes between PGLYRP1 and bacterially derived PGN, as well as multimerization of PGLYRP1 constitute potent ligands capable of binding TREM-1 and induce known TREM-1 mediated functions^9^.

Apart from the membrane-bound form, a soluble TREM-1 variant (sTREM-1) has been detected in body fluids. Several clinical studies reveal the presence of elevated sTREM-1 in ischemic^4^,^10^ as well as in infectious conditions. The level of sTREM-1 is significantly elevated in bronchoalveolar-lavage fluid from patients with pneumonia compared to patients without pneumonia^11^. Interestingly, high plasma sTREM-1 levels have been detected in sepsis and appear to be most helpful in differentiating patients with sepsis from those with systemic inflammatory response syndrome (SIRS), compared with other inflammatory markers like C-reactive protein and procalcitonin^12^. Increased serum levels of sTREM-1 can also be found in patients with clinical stable chronic obstructive pulmonary disease (COPD) and indicate a correlation between serum levels and disease severity^13^.

At present, there are two possible explanations for the origin of sTREM-1: Firstly translation of an alternative TREM-1 mRNA splice variant^14^ and secondly proteolytic cleavage (shedding) of mature, cell surface-anchored TREM-1^15^. In culture supernatants of lipopolysaccharides (LPS) stimulated neutrophils, TREM-1 surface expression is unchanged while sTREM-1 concentration is significantly increased. Moreover, the release of sTREM-1 is completely abrogated in the presence of cycloheximide, strongly suggesting that sTREM-1 is produced by *de novo* synthesis. However it is also possible that sTREM-1 might have been prestored intracellularly and requires the synthesis of other proteins in order to be released^16^. Nevertheless, there is also conclusive evidence in favor of the proteolytic mechanism of sTREM-1 generation. Gómez-Piña *et al.* detected no alternative splicing forms of TREM-1 in monocytes/macrophages. Moreover, metalloproteinase inhibitors increase the stability of TREM-1 surface expression, while significantly reducing sTREM-1 release in cultures of LPS-challenged human monocytes and neutrophils, indicating that metalloproteinases are responsible for shedding of the TREM-1 ectodomain through proteolytic cleavage^15^.

In summary, while the mechanisms of sTREM-1 generation are not completely clarified, there is convincing clinical data indicating a role for the presence of sTREM-1 as a relevant marker of inflammation in various diseases. However, whether the detection of sTREM-1 in body fluids provides reliable information of severity, particularly during infectious conditions (e.g. pneumonia and sepsis) is currently a matter of debate. At present, there are some additional restrictions to the use of sTREM-1 as an inflammatory marker due to difficulties in the comparability of various studies that find different levels of sTREM-1 concentrations, e.g. ranging in peripheral artery disease with CLI between 40 and 4,000 ng/mL^4^,^10^,^17^. Methodological aspects of sTREM-1 detection play a relevant role in this context, and general exogenous and endogenous interferences are possible factors that might contribute to an adequate detection of sTREM-1^18^ as well as sTREM-1 specific limitations like instability after repeated freeze/thaw cycles that degrade sTREM-1 may contribute to incorrect measurement^17^. In fact, discrepacies in sTREM-1 levels have been observed so far in previous clinical studies and may be related to differing study populations and analytical techniques. Several factors contributing to sTREM-1 assay variability have been described in the literature, including the substrate used in the final end-point reaction as with any Western blot or immunoblot systems, the antigen (standard) used as calibrator, and the nature and avidity of the primary antibody^19^. In early days of sTREM-1 research individual home-made immunoblot techniques and enzyme immunoassays have been employed^11^,^20^,^21^. As a feasible, timesaving and comparatively cheap method ELISA technique has been increasingly used for the detection of sTREM-1, especially during the last years after commercial ELISA kits have become available. Since then the ELISA technique has evolved as the preferred method of detection by many investigators in the TREM-1 field in a broad range of disease^15^,^22^,^23^,^24^.

In this study we examined serum interferences in three different sTREM-1 ELISAs and compared their ELISA specific characteristics like accuracy, precision and limit of detection (LOD). We determined factors affecting the measurement of sTREM-1 by ELISA that may therefore elucidate the variations of sTREM-1 levels in the published literature.

## Results

### Comparison of three sTREM-1-specific ELISAs

To identify individual characteristics of different sTREM-1 ELISAs we tested and directly compared the following assays: one previously established self-made ELISA marked Radsak^13^, a commercially available kit from R&D Systems - performed twice with different buffers (pcb = protein-containing buffer, and buffer without protein) and one ELISA kit by Hycult Biotech. Reference concentration and standard dilution series are important factors that can explain general discrepancies in the comparability of studies from different investigators as well as for defining general threshold values of diagnostic tools. Therefore we generated reference curves of each of the employed ELISAs. 2,000 pg/mL rh-sTREM-1 was employed and twofold serially diluted for curve creation. Primary ELISA data (optical density (OD) values) were processed via GraphPad Prism version 5 and Microsoft Excel 2008 calculation by four-parameter logistic fitting of the reference curve *y* = *(A* − *D)*/*[1* + *(x*/*C)*^*B*^] + *D* where *A* = OD_min_ (minimal optical density), *B* = slope, *C* = EC50 (half maximal effective concentration) and *D* = OD_max_ (maximal optical density)^25^.

**Figure 1:**
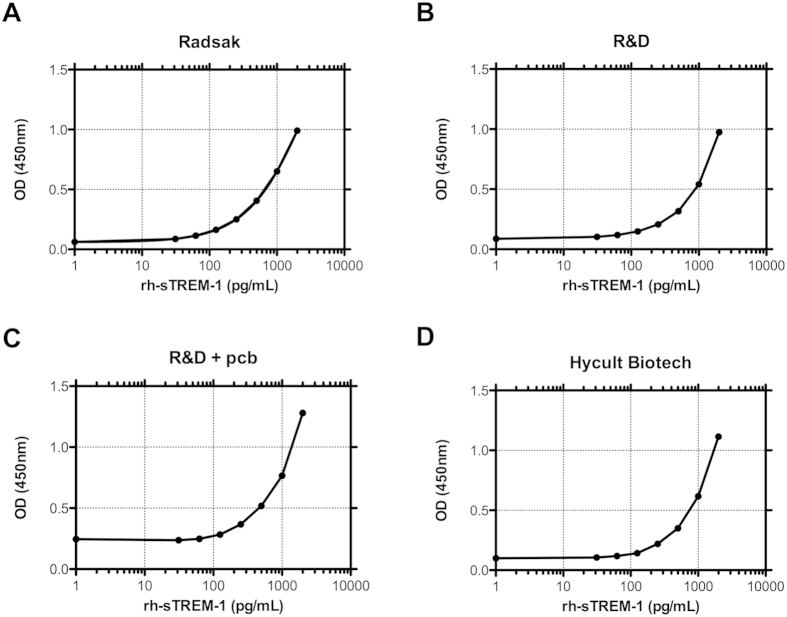
Comparison of sTREM-1 specific ELISAs. Comparison of curve progression of rh-sTREM-1 standard in sTREM-1 Radsak (**A**), R&D (**B**), R&D + pcb (**C**) and Hycult Biotech (**D**) ELISA. 2,000 pg/mL of appropriate rh-sTREM-1 was employed and further twofold serially diluted for each ELISA. The OD was measured and depicted as the mean value of duplicates. One representative experiment out of three repeats is shown. The curves follow a four-parametric nonlinear curve fitting.

Figure 1 shows that all dilution series of the ELISA specific rh-sTREM-1 reveal a typical sigmoid curve course in the semi-log plot. The four-parametric nonlinear regression resulted in good correlation (R^2^ = 1) and comparable high slopes (Radsak 0.9602, R&D 1.011, R&D + pcb 1.060, Hycult Biotech 1.134) for all ELISAs.

Accuracy and precision of investigated sTREM-1 ELISAs were determined by standard addition comprising serial dilutions of the reference standard (recombinant human (rh) sTREM-1) in dilution buffer. The precision of an ELISA is generally defined by its degree of variability whereupon the total variability can be estimated from the same experiment (intra-assay variability) in combination with replicate values of several independent experiments following identical procedure (inter-assay variability). Therefore each ELISA was performed multiple times and sTREM-1 concentrations were calculated of measured OD by the above described formula. As shown in Table 1, the different sTREM-1 ELISAs showed overall high precision, not exceeding a coefficient of variation (CV) of 20% (Radsak 15%, R&D 13.6%, R&D + pcb 20%, Hycult Biotech 9%) calculated for total of the serially diluted concentrations. The recovery was evaluated by addition of human serum of healthy donors 1:4 diluted and once more addition and serially dilution of rh-sTREM-1. The overall recovery was expected 100% if the addition of serum had no influence on sTREM-1 detection, indicating accurate results in terms of absolute quantity and exclusion of matrix effects. As shown in Table 1 calculation of recovery revealed large differences between the investigated ELISAs, ranging from 40 to 84% (Radsak 58.5%, R&D 49.3%, R&D + pcb 40.6%, Hycult Biotech 84.1%). The LOD was determined based on blank measurements and the formula LOD =  mean +3x standard deviation (SD) resulting in 4-195 pg/mL depending on the ELISA and engaged buffers (Radsak 4.0 pg/mL, R&D 23.2 pg/mL, R&D + pcb 194.6 pg/mL, Hycult Biotech 143.4 pg/mL) (Table 1).

**Figure 2:**
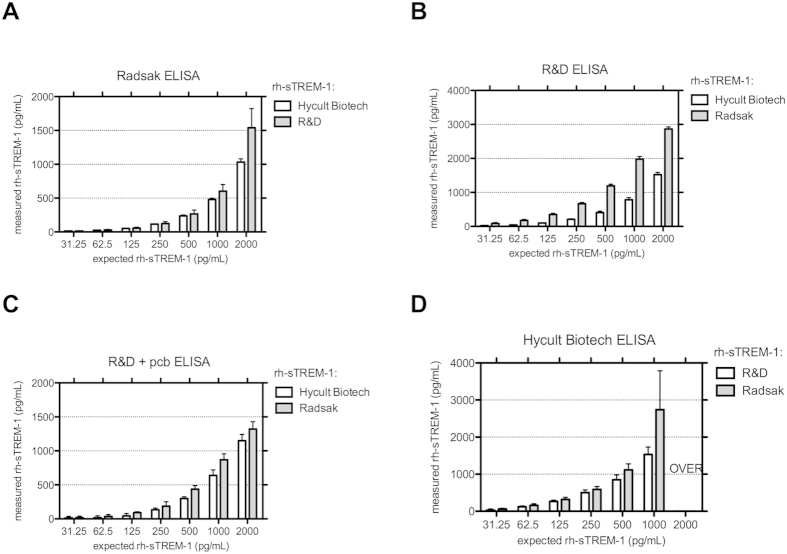
Different ELISAs and rh-sTREM-1 concentrations were directly compared. Radsak (**A**), R&D (**B**), R&D + pcb (**C**) and Hycult Biotech (**D**) sTREM-1 ELISA were investigated for recovery of 2,000 pg/mL rh-sTREM-1 and twofold dilution series. Data are expressed as mean and SD conducted in duplicates and three independent experiments. Values ranging beyond the respective reference curve were set to zero for graph creation and excluded of further calculations or marked OVER.

To directly compare currently available ELISAs we tested individual rh-sTREM-1 in each of the different assays. rh-sTREM-1 serially dilution was again performed as described above. Investigating the recovery of different rh-sTREM-1 in each ELISA discrepancies in concentrations were detected with overall highest amount in our self-made ELISA, followed by R&D and Hycult Biotech (Fig. 2). Notably the performance of the R&D ELISA was again dependent on the used buffers with higher concentrations detected in protein-containing conditions. Furthermore large differences in concentrations of investigated rh-sTREM-1 were detected in the Hycult Biotech ELISA compared to the other rh-sTREM-1. As shown in Fig. 2., Radsak rh-sTREM-1 2,000 pg/mL concentration was not ascertainable by four-parameter logistic fitting because of values ranging above the OD_max_ of the reference curve of the Hycult Biotech ELISA, therefore these values were excluded from graph calculation.

In conclusion, all investigated sTREM-1 ELISAs showed individual characteristics in their performance. Summing up, Radsak ELISA is able to detect the lowest levels of sTREM-1 when using our own rh-sTREM-1 as reference standard, whereas Hycult Biotech ELISA showed best performance in serum containing conditions. Performance of R&D ELISA was extremely dependent on inserted buffers with generally better results without protein containing buffer.

### Human serum contains a heat-sensitive confounding factor for the detection of rh-sTREM-1

**Figure 3:**
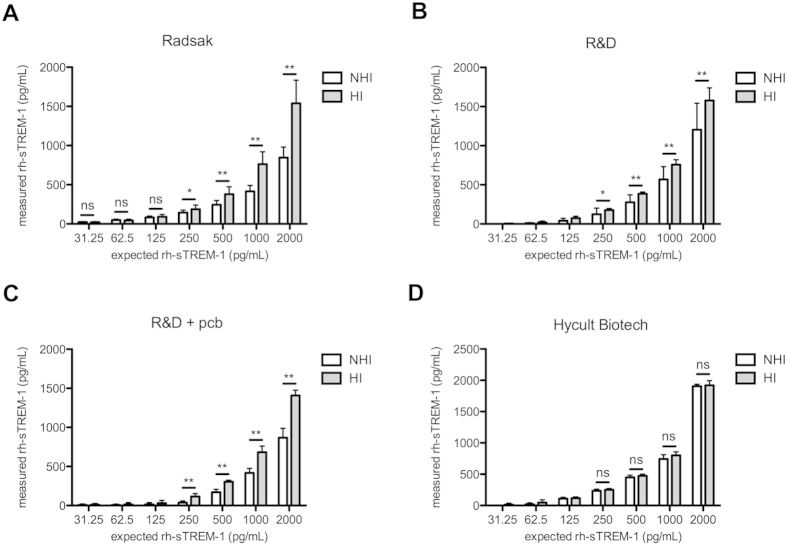
Effect of serum heat-inactivation on the detection of rh-sTREM-1 was assessed in different ELISAs. NHI and HI sera were diluted 1:4 and 2,000 pg/mL rh-sTREM-1 was added. Twofold serially dilution was performed and rh-sTREM-1 concentration was measured by the indicated ELISA. Mean and SD for three independent experiments each conducted in duplicates are shown. Values ranging beyond the designed standard curve were set to zero for graph creation and excluded of further calculations. Data were statistical tested for significant differences of rh-sTREM-1 of indicated concentrations in NHI and HI serum by two-tailed Wilcoxon matched-pairs test, followed by Bonferroni correction for multiple testing. *p < 0.05, **p < 0.01.

Evaluating accuracy of indicated ELISAs serum interferences are assumed regarding recovery rates between 40 and 84%. To further investigate these interferences in defined sTREM-1 concentrations, we examined the effect of human serum from healthy donors and furthermore the effect of heat-inactivation^26^,^27^,^28^ on detection of rh-sTREM-1 and dilution series in the different ELISAs. NHI and HI serum samples were employed at 1:4 dilutions and 2,000 pg/mL rh-sTREM-1, further 1:2 diluted in appropriate buffers. Figure 3A confirms the accuracy results of the Radsak ELISA, where only about half of the applied sTREM-1 standard amount can be detected after the addition of NHI serum. Aside in samples containing HI serum sTREM-1 standard was at a significantly higher amount detectable, indicating a positive effect of heat-inactivation of serum on sTREM-1 detection in a concentration dependent manner (2,000 pg/mL p = 0.005, 1,000 pg/mL p = 0.005, 500 pg/mL p = 0.005, 250 pg/mL p = 0.010, 125 pg/mL p = 0.094, 62.5 pg/mL p = 0.094, 31.25 pg/mL p = 0.115). Similar results were obtained when NHI and HI sera were engaged in R&D ELISA with pcb (2,000 pg/mL p = 0.005, 1,000 pg/mL p = 0.005, 500 pg/mL p = 0.005, 250 pg/mL p = 0.026, 125 pg/mL excluded, 62.5 pg/mL excluded, 31.25 pg/mL excluded) (Fig. 3B), with slight improvement of the performance when surrendering pcb (2,000 pg/mL p = 0.005, 1,000 pg/mL p = 0.005, 500 pg/mL p = 0.005, 250 pg/mL p = 0.005, 125 pg/mL excluded, 62.5 pg/mL excluded, 31.25 pg/mL excluded) (Fig. 3C). In contrast, performance of Hycult Biotech ELISA was completely independent of these heat-sensitive confounding factors (2,000 pg/mL p = 0.156, 1,000 pg/mL p = 0.063, 500 pg/mL p = 0.094, 250 pg/mL p = 0.156, 125 pg/mL excluded, 62.5 pg/mL excluded, 31.25 pg/mL excluded) (Fig. 3D). OD values ranging below the calculated OD_min_ had to be excluded of further calculations. Apart from that, the detectable differences between HI and NHI sera were only significant for several dilutions with correspondent high rh-sTREM-1 standard concentrations in the Radsak ELISA (Fig. 3A).

### Complement Interferes in sTREM-1 ELISA

**Figure 4:**
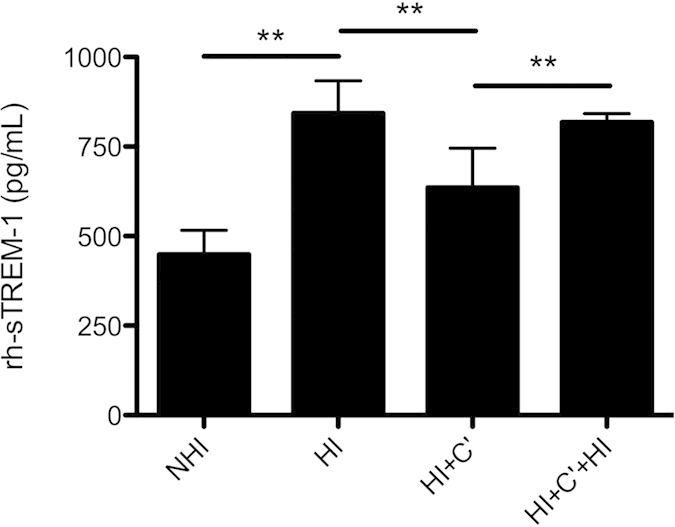
Complement interferes in sTREM-1 ELISA. NHI and HI sera were 1:4 diluted and 4 U of reconstituted guinea pig complement (C’) was added to 240 μL-diluted sera. HI serum after addition of C’ was again heat-inactivated at 56 °C for 30 min to inactivate the complement. 2,000 pg/mL rh-sTREM-1 was added to each preparation and twofold serially dilution was performed. Shown are the results of extrapolated rh-sTREM-1 at 1,000 pg/mL tested by Radsak sTREM-1 ELISA. Mean and SD for three independent experiments each conducted in duplicates are shown. Statistical significance for indicated samples was calculated by two-tailed Wilcoxon matched-pairs test followed by Bonferroni correction for multiple testing. **p < 0.01.

Complement components present in serum are known to react with several immunoglobulins^29^ and interference with detection in immunoassay can partly be eliminated by serum heat-inactivation^26^. Therefore rh-sTREM-1 concentration was measured in 1:4 diluted NHI and HI serum, as well as after the addition of guinea pig complement and re-heat-inactivation^26^ employing Radsak sTREM-1 ELISA. These experiments were again performed in 1:2 serially dilution of rh-sTREM-1. As shown above rh-sTREM-1 in NHI serum resulted in significantly increased recovery (p = 0.005) compared to HI serum (Fig. 4). Furthermore 4 U exogenous guinea pig complement was added to the 1:4 dilution of HI serum. Addition of complement resulted in significantly decreased rh-sTREM-1 concentration (p = 0.005) demonstrating that the positive effect of heat-inactivation on rh-sTREM-1 detection compared to NHI serum can be effaced through the addition of complement. To further test if re-heat-inactivation of HI serum with complement will restore measured rh-sTREM-1 amount we re-heat-inactivated HI serum with complement. Interestingly, rh-sTREM-1 after heat-inactivation of HI serum with complement was again significantly increased as compared to non heat-inactivated HI serum with complement (p = 0.005) (Fig. 4).

## Discussion

sTREM-1 is currently debated as a diagnostic biomarker as well as to prognosticate the outcome of a septic patient by determination of its concentration^30^. Early publications used immunoblot techniques for the detection of sTREM-1^11^,^31^ but since 2005 most published studies use commercially available or self-made sandwich ELISAs^13^,^32^,^33^. Objective analysis of the published literature is difficult because of a huge heterogeneity among studies. Most importantly, the techniques used to measure the sTREM-1 concentrations are not always comparable with large variations both during the preanalytical and the analytical period. Some commercial kits have been withdrawn from the market during the last years due to unreliable results^17^,^30^ and huge differences in the detected concentration under similar pathological conditions have been described^4^. Therefore the aim of this study was to evaluate currently available sTREM-1 ELISAs and explore confounding factors. Investigated ELISAs include one in-house developed sTREM-1 ELISA referred to as Radsak^13^, one from R&D Systems, tested with and without protein containing buffers, and one from Hycult Biotech. Overall the investigated sTREM-1 ELISAs revealed individual characteristics in precision, accuracy and LOD. Employing different buffer compositions in R&D ELISA, performance was extremely dependent on protein content with generally better results without protein containing buffer (Table 1). Especially when testing different rh-sTREM-1 concentrations in above-named ELISAs, the recovery rates of different rh-sTREM-1 varied and performance of the R&D ELISA was again dependent on the used (protein-containing) buffers. This effect may be due to the influence of the matrix on rh-sTREM-1 detection due to adsorption without a carrier protein to the tubes used for serial dilution^34^ and also changes in the chemical structure in terms of redox forms of the standard can influence the performance in the ELISA^35^. Generally, rh-sTREM-1 concentrations differed in the investigated ELISAs revealing poor variability between the different kits. This may be due to the lack of standardization in determining protein content but further parameters like engaged buffers seem to be involved since measured concentrations of the same rh-sTREM-1 are disproportional between different assays (Fig. 2). Furthermore, Hycult Biotech ELISA showed good performance in serum containing conditions (recovery of 84%) and also the best linear dilution indicative of less matrix effects. In contrast, the other tested ELISAs showed lower recovery rates (about 50%) that indicate interference of serum compounds in these assays. Serum heat-inactivation has been shown to be beneficial in improving target detection when optimizing immunoassay conditions^26^,^27^,^28^. Therefore rh-sTREM-1 detection was evaluated in the presence of NHI and HI serum and a heat sensitive confounding factor in human serum with different susceptibility of the investigated ELISAs was revealed. rh-sTREM-1 detection was obviously improved after HI of serum. Notably the detectable differences between HI and NHI sera were only significant for several dilutions with high rh-sTREM-1 concentrations in the Radsak ELISA (Fig. 3). Therefore improved detection of sTREM-1 through dilution of serum could be assumed, nevertheless increased serum dilution can also lead to decreased sensitivity^18^. Focusing on the recovery rates and effects of heat-inactivation, we observed analogue results between Radsak and R&D ELISA. One similarity between those two assays is the polyclonal detection antibody. Because the exact contents of the Hycult Biotech kit are not provided by the manufacturer, we cannot exclude a distinction at this point. The same applies for all further contents of this specific assay so that we have to assume different ingredients of the reagents.

Complement components present in serum are known to react with several immunoglobulins and therefore act as interfering factors^29^ whereat heat-inactivation revealed to be an effective procedure to eliminate complement^26^. Hence complement as a heat-sensitive confounding factor in specific sTREM-1 ELISA was confirmed by the addition of complement and re-heat-inactivation (Fig. 4). As far as we know special sources of error were not yet defined for sTREM-1 ELISA but well-known general exogenous and endogenous interferences are possible factors. Particularly endogenous factors are difficult to detect and eliminate, since they vary from patient to patient and also from time to time in one patient. Amongst others these endogenous factors include hyperlipidemia and nonesterified fatty acids, crossreacting heterophilic anti-immunoglobulin antibodies and complement^18^. In addition, differences in sample logistics are well-known to have an impact on the outcome of assays, especially for biomarkers that are frequently influenced by complement. Standardization of sample collection is therefore advisable. Furthermore, sTREM-1 specific limitations like instability after repeated freeze/thaw cycles may impair correct measurement^17^.

We conclude that exogenous and endogenous interferences in sTREM-1 ELISA make it still difficult to compare data of several investigators in particular if different ELISAs were used. Therefore sTREM-1 ELISA has to be further improved until capable for clinical application and comparable studies. Heat-inactivation may be an effective mechanism for reducing complement as a confounding factor. Of the tested assays, the Hycult Biotech ELISA is less hampered by the complexity of the sample types. Nevertheless studies on established patient samples need to be performed to confirm our results.

## Materials and Methods

### Study subjects, peripheral blood collection, and processing

All human studies were performed in accordance with the Declaration of Helsinki and were approved by the Landesaerztekammer Rhineland-Palatine Ethics Committee according to the institutional guidelines. Written informed consent was obtained from healthy donors before sample collection and peripheral blood was collected by venipuncture into serum vacutainers (Sarstedt AG & Co., Nümbrecht, Germany). Samples were kept at room temperature (RT) for about 15 min and afterwards centrifuged at 2,000 rpm for 10 min. For heat-inactivation samples were incubated at 56 °C for 30 min.

### Measurement of sTREM-1 concentration by ELISA

#### sTREM-1 ELISA by Radsak

This self-made sTREM-1 ELISA has been established and described previously^13^. For the detection of sTREM-1, 50 μL of anti-TREM-1 (clone 6B1.1G12 mAb) was coated at 10 μg/mL in coating buffer (Na_2_HPO_4_ × 2H_2_O 0.1 M pH = 9.3) at 4 °C over night and 37 °C for one hour respectively. Then plates were blocked with 200 μL blocking buffer (PBS 1%, BSA 1%) for 1.5 hours at RT. Afterwards the standard (recombinant human TREM-1 in 7.5% BSA-PBS) and the samples were added and the plates were incubated for 1.5 hours at RT. For analysis of sera samples, sera were diluted as indicated prior to addition to the plates (100 μL/well). After incubation for 1.5 hours plates were washed and the biotinylated detection polyclonal Ab anti-TREM-1 (R&D Systems Europe, Abingdon, UK) at 5 μg/mL was added for 1 hour at RT. Plates were then washed and streptavidine-HRP (R&D Systems, Europe, Abingdon, UK) was added for 20 min at RT. Plates were washed again, they were developed using the Tetramethylbenzidine Peroxidase Substrate System (KPL, Gaithersburg, Md, USA) and reaction was stopped by addition of H_2_SO_4_. All dilutions were carried out in blocking buffer. The absorbance was measured at 450 nm.

#### Human TREM-1 DuoSet by R&D

Human TREM-1 DuoSet (R&D Systems Europe, Abingdon, UK) was performed according to manufacturer’s instruction. When indicated buffer solutions where replaced by buffers (coating and blocking buffer = pcb = protein containing buffer) described above at Radsak ELISA.

#### Human sTREM-1 ELISA kit by Hycult Biotech

Human sTREM-1 ELISA (HK348) was performed according to manufacturer’s instruction using pre-coated plates (Hycult Biotech, Uden, Netherlands).

### Complement Addition to HI Serum

Complement addition to heat-inactivated (HI) serum was performed as described by Namekar *et al.*^26^. HI serum was diluted 1:4 in blocking buffer and 4 U of reconstituted guinea pig complement (C’) (Sigma-Aldrich GmbH, Germany) were added to 240 μL of diluted serum. In separate samples, HI serum after addition of C’ was again heat-inactivated at 56 °C for 30 min to inactivate the complement. NHI, HI, HI serum with C’ and re-heat-inactivated HI serum with C’ were tested by Radsak sTREM-1 ELISA.

### Statistical Analysis

Calculations for mean values, SD and reference curve parameters were performed with GraphPad Prism5 software (San Diego, CA, USA) and Microsoft Excel 2008 (Redmond, WA, USA). Statistical significance was calculated by two-tailed Wilcoxon matched-pairs tests followed by Bonferroni correction for multiple testing. Statistical significance was assumed for p < 0.05. If values ranged beyond the designed reference curve they had to be excluded of graphs and further calculations.

## Additional Information

**How to cite this article**: Hasibeder, A. *et al.* Evaluation and Validation of the Detection of soluble Triggering Receptor Expressed on Myeloid Cells 1 by Enzyme-linked immunosorbent Assay. *Sci. Rep.* **5**, 15381; doi: 10.1038/srep15381 (2015).

## Figures and Tables

**Table 1:** Precision, accuracy and LOD was determined for different ELISAs.

	Total variability (%)	Accuracy (%)	Limit of detection (pg/mL)
Radsak	15.0	58.5	4.0
R&D	13.6	49.3	23.2
R&D + pcb	20.0	40.6	194.6
Hycult Biotech	9.0	84.1	143.4

2,000 pg/mL rh-sTREM-1 was employed and further twofold serially diluted in four ELISAs (Radsak, R&D, R&D + pcb and Hycult Biotech). Precision of rh-sTREM-1 determination was carried out by total variability. For accuracy 1:4 diluted human serum was added to initial rh-sTREM-1 concentration. The OD was measured and the CV calculated for total concentrations. LOD was determined based on blank measurements and the formula LOD = 3 × SD + mean. Each ELISA was performed in duplicates and repeated three times, indicated values represent the mean. Values ranging beyond the designed standard curve were excluded of further calculations.

## References

[b1] Bouchon A., Dietrich J. & Colonna M. Cutting edge: Inflammatory responses can be triggered by TREM-1, a novel receptor expressed on neutrophils and monocytes. J Immunol 164, 4991–4995 (2000). 10799849 10.4049/jimmunol.164.10.4991

[b2] Bouchon A., Facchetti F., Weigand M. A. & Colonna M. TREM-1 amplifies inflammation and is a crucial mediator of septic shock. Nature 410, 1103–1107 (2001). 11323674 10.1038/35074114

[b3] Mohamadzadeh M. *et al.* Activation of triggering receptor expressed on myeloid cells-1 on human neutrophils by marburg and ebola viruses. J Virol 80, 7235–7244 (2006). 16809329 10.1128/JVI.00543-06 PMC1489070

[b4] Dopheide J. F. *et al.* Critical limb ischaemia is characterised by an increased production of whole blood reactive oxygen species and expression of TREM-1 on neutrophils. Atherosclerosis 229, 396–403 (2013). 23880194 10.1016/j.atherosclerosis.2013.05.029

[b5] Kuai J. *et al.* TREM-1 expression is increased in the synovium of rheumatoid arthritis patients and induces the expression of pro-inflammatory cytokines. Rheumatology (Oxford) 48, 1352–1358 (2009). 19713442 10.1093/rheumatology/kep235

[b6] Schenk M., Bouchon A., Seibold F. & Mueller C. TREM-1–expressing intestinal macrophages crucially amplify chronic inflammation in experimental colitis and inflammatory bowel diseases. J Clin Invest 117, 3097–3106 (2007). 17853946 10.1172/JCI30602 PMC1974863

[b7] Saurer L. L. *et al.* Elevated levels of serum-soluble triggering receptor expressed on myeloid cells-1 in patients with IBD do not correlate with intestinal TREM-1 mRNA expression and endoscopic disease activity. J Crohns Colitis 6, 913–923 (2012). 22410349 10.1016/j.crohns.2012.02.010

[b8] Haselmayer P., Grosse-Hovest L., Landenberg, von P., Schild H. & Radsak M. P. TREM-1 ligand expression on platelets enhances neutrophil activation. Blood 110, 1029–1035 (2007). 17452516 10.1182/blood-2007-01-069195

[b9] Read C. B. *et al.* Cutting Edge: Identification of Neutrophil PGLYRP1 as a Ligand for TREM-1. The Journal of Immunology (2015). 10.4049/jimmunol.1402303 PMC4319313 25595774

[b10] Dopheide J. F. *et al.* Change of walking distance in intermittent claudication: impact on inflammation, oxidative stress and mononuclear cells: a pilot study. Clin Res Cardiol (2015). 10.1007/s00392-015-0840-5 25772524

[b11] Gibot S. *et al.* Soluble triggering receptor expressed on myeloid cells and the diagnosis of pneumonia. N Engl J Med 350, 451–458 (2004). 14749453 10.1056/NEJMoa031544

[b12] Gibot S. & Cravoisy A. Soluble form of the triggering receptor expressed on myeloid cells-1 as a marker of microbial infection. Clin Med Res 2, 181–187 (2004). 15931355 10.3121/cmr.2.3.181 PMC1069091

[b13] Radsak M. P. *et al.* Soluble triggering receptor expressed on myeloid cells 1 is released in patients with stable chronic obstructive pulmonary disease. Clin. Dev. Immunol. 2007, 52040 (2007). 18317529 10.1155/2007/52040 PMC2246041

[b14] Gingras M.-C., Lapillonne H. & Margolin J. F. TREM-1, MDL-1, and DAP12 expression is associated with a mature stage of myeloid development. Mol Immunol 38, 817–824 (2002). 11922939 10.1016/s0161-5890(02)00004-4

[b15] Gómez-Piña V. *et al.* Metalloproteinases shed TREM-1 ectodomain from lipopolysaccharide-stimulated human monocytes. J Immunol 179, 4065–4073 (2007). 17785845 10.4049/jimmunol.179.6.4065

[b16] Mahdy A. M., Lowes D. A., Galley H. F., Bruce J. E. & Webster N. R. Production of soluble triggering receptor expressed on myeloid cells by lipopolysaccharide-stimulated human neutrophils involves de novo protein synthesis. Clin. Vaccine Immunol. 13, 492–495 (2006). 16603617 10.1128/CVI.13.4.492-495.2006 PMC1459640

[b17] Gibot S. Soluble triggering receptor expressed on myeloid cells-1 and diagnosis of ventilator-associated pneumonia. Intensive Care Med 35, 1644–author reply 1645–6 (2009). 10.1007/s00134-009-1547-8 19543708

[b18] Weber T. H., Käpyaho K. I. & Tanner P. Endogenous interference in immunoassays in clinical chemistry. A review. Scand. J. Clin. Lab. Invest. Suppl. 201, 77–82 (1990). 2244186

[b19] Phua J. *et al.* Soluble triggering receptor expressed on myeloid cells-1 in acute respiratory infections. Eur. Respir. J. 28, 695–702 (2006). 16837506 10.1183/09031936.06.00005606

[b20] Tzivras M. *et al.* Role of soluble triggering receptor expressed on myeloid cells in inflammatory bowel disease. World J. Gastroenterol. 12, 3416–3419 (2006). 16733861 10.3748/wjg.v12.i21.3416 PMC4087875

[b21] Routsi C. *et al.* Does soluble triggering receptor expressed on myeloid cells-1 play any role in the pathogenesis of septic shock? Clin Exp Immunol 142, 62–67 (2005). 16178857 10.1111/j.1365-2249.2005.02887.x PMC1809490

[b22] Chao W.-C. *et al.* Predictive value of serial measurements of sTREM-1 in the treatment response of patients with community-acquired pneumonia. J. Formos. Med. Assoc. 106, 187–195 (2007). 17389162 10.1016/S0929-6646(09)60239-4

[b23] Bucova M. *et al.* Inflammatory Marker sTREM-1 Reflects the Clinical Stage and Respiratory Tract Obstruction in Allergic Asthma Bronchiale Patients and Correlates with Number of Neutrophils. Clin. Dev. Immunol. 2012, 1–8 (2012). 10.1155/2012/628754 PMC3399449 22829716

[b24] Carrol E. D. *et al.* The diagnostic and prognostic accuracy of five markers of serious bacterial infection in Malawian children with signs of severe infection. - PubMed - NCBI. PLoS ONE 4, e6621 (2009). 19675669 10.1371/journal.pone.0006621 PMC2721152

[b25] Dehus O. *et al.* Development and in-house validation of an allergen-specific ELISA for quantification of Bet v 4 in diagnostic and therapeutic birch allergen products. Anal Bioanal Chem 407, 1673–1683 (2015). 25572690 10.1007/s00216-014-8418-z

[b26] Namekar M., Kumar M., O’Connell M. & Nerurkar V. R. Effect of Serum Heat-Inactivation and Dilution on Detection of Anti-WNV Antibodies in Mice by West Nile Virus E-protein Microsphere Immunoassay. PLoS ONE 7, e45851 (2012). 23049879 10.1371/journal.pone.0045851 PMC3457982

[b27] Dias D. *et al.* Optimization and validation of a multiplexed luminex assay to quantify antibodies to neutralizing epitopes on human papillomaviruses 6, 11, 16, and 18. Clin. Diagn. Lab. Immunol. 12, 959–969 (2005). 16085914 10.1128/CDLI.12.8.959-969.2005 PMC1182182

[b28] Schnaidt M. *et al.* HLA antibody specification using single-antigen beads–a technical solution for the prozone effect. Transplantation 92, 510–515 (2011). 21869744 10.1097/TP.0b013e31822872dd

[b29] Papp K. *et al.* On-chip complement activation adds an extra dimension to antigen microarrays. Mol. Cell Proteomics 6, 133–140 (2007). 17071944 10.1074/mcp.T600036-MCP200

[b30] Lemarié J., Barraud D. & Gibot S. Host response biomarkers in sepsis: overview on sTREM-1 detection. Methods Mol Biol 1237, 225–239 (2015). 25319790 10.1007/978-1-4939-1776-1_17

[b31] Gibot S. *et al.* A soluble form of the triggering receptor expressed on myeloid cells-1 modulates the inflammatory response in murine sepsis. J Exp Med 200, 1419–1426 (2004). 15557347 10.1084/jem.20040708 PMC2211948

[b32] Determann R. M. *et al.* Serial changes in soluble triggering receptor expressed on myeloid cells in the lung during development of ventilator-associated pneumonia. Intensive Care Med 31, 1495–1500 (2005). 16195904 10.1007/s00134-005-2818-7

[b33] Giamarellos-Bourboulis E. J. *et al.* Soluble triggering receptor expressed on myeloid cells 1 as an anti-inflammatory mediator in sepsis. Intensive Care Med 32, 237–243 (2006). 16450102 10.1007/s00134-005-0017-1

[b34] Bratcher P. E. & Gaggar A. Factors influencing the measurement of plasma/serum surfactant protein D levels by ELISA. PLoS ONE 9, e111466 (2014). 25365324 10.1371/journal.pone.0111466 PMC4218753

[b35] Lundberg M. *et al.* Methodological Aspects of ELISA Analysis of Thioredoxin 1 in Human Plasma and Cerebrospinal Fluid. PLoS ONE 9, e103554 (2014). 25075746 10.1371/journal.pone.0103554 PMC4116216

